# Coffee Cherry Pulp by-Product as a Potential Fiber Source for Bread Production: A Fundamental and Empirical Rheological Approach

**DOI:** 10.3390/foods10040742

**Published:** 2021-04-01

**Authors:** Gustavo Armando Rosas-Sánchez, Zorba Josué Hernández-Estrada, Mirna Leonor Suárez-Quiroz, Oscar González-Ríos, Patricia Rayas-Duarte

**Affiliations:** 1 Tecnológico Nacional de México/IT de Veracruz, Calz. Miguel Ángel de Quevedo 2779, Col. Formando Hogar, 91860 Veracruz, Mexico; armando.rosg@gmail.com (G.A.R.-S.); mirna.sq@veracruz.tecnm.mx (M.L.S.-Q.); oscar.gr@veracruz.tecnm.mx (O.G.-R.); 2 Robert M. Kerr Food and Agricultural Products Center, Biochemistry and Molecular Biology Department, Oklahoma State University, Stillwater, OK 74078, USA

**Keywords:** coffee pulp, by-products, rheological characterization, viscoelasticity, creep recovery, wheat, gluten

## Abstract

Effects of substituting of wheat flour with coffee cherry pulp powder (CCPP) (coffee by-product as fiber source) at 0, 1.2, 2.3, and 4.7% dry basis (0, 1.25, 2.5, and 5% wet basis) on dough and gluten rheological properties and baking quality were investigated. Rheological properties were analyzed during mixing, compression recovery, and creep-recovery. A rheological approach was adopted to study the viscoelasticity of dough enriched with fiber. The data obtained were analyzed with the Kelvin–Voigt model and the parameters were correlated to bread volume and crumb firmness to assess the effect of incorporating CCPP. A decrease in gluten’s elastic properties was attributed to the water-binding and gelling properties of CCPP. Stiffness of dough and crumb firmness increased as the level of CCPP increased and bread volume decreased. Stiffer dough corresponded with lower compliance values and higher steady state viscosity compared to the control. A follow-up study with 5% CCPP and additives is recommended to overcome the reduction in elastic recovery and bread volume.

## 1. Introduction

Coffee is a popular beverage and arguably one of the most traded commodities in the world. Due to the high demand for coffee, large amounts of by-products such as pulp, silver skin, and parchment are generated. According to the International Coffee Organization, in 2019, about 10 million tons of fresh coffee were produced globally. It implies the generation of approximately 3 million tons of coffee cherry pulp (CCP) as a by-product and represents a severe environmental problem. Additionally, CCP contains fiber, minerals, amino acids, and polyphenolic compounds potentially beneficial for human nutrition [1]. Several studies have analyzed CCP’s chemical composition, reporting that the dried material has about 10% crude protein, 21% crude fiber, 8% ash, and 44% nitrogen-free extract; these values change according to coffee variety, location, and agricultural practices [2]. Furthermore, hydroxycinnamic acids as chlorogenic, caffeic, and ferulic acid in CCP are of interest for their antioxidant properties, which neutralize excess free radicals to prevent cell damage from free radicals [3,4].

On the other hand, bakery products are highly consumed worldwide, usually elaborated with refined flours, deficient dietary fiber, and other nutrients [5]. So, CCP could be used as an ingredient in fiber-deficient bakery products. Bakery products are technologically demanding, and changes in their formulation or process could affect their quality. Fiber modifies the rheological properties of dough and bread quality [6]. It is well known that the food matrix’s viscoelasticity or rheological behavior is related to the composition, structure, and stability; therefore, the rheological characterization of a food matrix and its components is overriding for predicting food quality [7].

In the breadmaking process, the dough is subjected to different deformation types during mixing, fermentation, rolling and shaping, proofing, and baking [8], and even when we chew it. This is why, rheological studies are helpful research tools to determine the dough’s behavior and estimate the interrelations between flour composition, ingredients functionality, process parameters, and loaf characteristics [8].

Empirical tests conducted with the farinograph, alveograph, and extensograph are widely used in the baking industry as tools to predict baking quality while fundamental rheological tests continue probing it [9,10,11,12,13]. The creep recovery test measures the material’s viscoelasticity by applying constant stress during a determined time [14]. Many researchers have tried to understand gluten and dough’s viscoelastic behavior by modeling experimental data using the Burgers model with Maxwell and Kelvin elements [10,12,13,15,16,17]; the model’s values were correlated with breadmaking quality [6,13]. Because of the complexity of the protein network in gluten, the viscoelastic behavior for a breadmaking quality can change among wheat cultivars, new bread formulations, and storage.

Creep and recovery tests have been widely used to measure the impact of dietary fiber on the viscoelastic behavior of rice flour doughs [18], the effect of high molecular weight glutenin subunits in gluten and its relationship with the quality of bread [13], rheological, and functional properties of red bean flour of gluten-free batter for cupcakes [17]. Other authors investigated the effect of flour substitution by bran with different particle sizes on dough rheology. They reported a significant effect of substitution level and a minor effect of particle size [19]. In general, fiber added to flour affects the gluten-starch interaction, reduces swelling of starch granules and its availability for gelatinization, compels gas cells to expand, and adversely affects dough viscoelastic behavior constraining dough availability to trap gas [20]. It is of interest to assess the CCP by-product in food applications and specifically in baked products. By assessing the influence of CCPP in gluten and non-gluten components of dough, a comparison can be made on the changes in dough’s viscoelasticity and stiffness, both essential attributes in the baking process [13]. Thus, the objective of this study was to investigate the effect of CCPP on the wheat dough and gluten rheological properties and baking quality without any other additives.

## 2. Materials and Methods

### 2.1. Materials

Decaffeinated CCPP (Pulphari) was obtained from Grupo Techver S.A. de C.V. (Veracruz, Mexico). Commercial Spring wheat flour (enriched, bleached) was donated by Shawnee Milling Company (Shawnee, OK, USA).

The particle size of CCPP was further reduced with an impact-type mill (Kitchen Mill, Blendtec, Orem, UT, USA), sieved (screen number 100) to obtain a more homogeneous powder then stored in closed containers at room temperature until needed for analyses. In total, three levels of wheat flour substitution by CCPP were used 0, 1.25, 2.5, and 5% wet basis (0, 1.2, 2.3, and 4.7% dry basis). These levels were selected from results of preliminary experiments with levels up to 30% CCPP. At CCPP substitution levels greater than 5%, the bread had a grassy and earthy aftertaste, and in samples with substitution levels greater than 10%, the dough was difficult to handle.

### 2.2. Flour and CCPP Analysis

CCPP and wheat flour were evaluated according to the Association of Official Agricultural Chemists (now AOAC International) Official Methods [21] for protein (920.87), moisture (925.10), ash (923.03), and fat (945.16). Soluble and insoluble dietary fiber were evaluated by the American Association of Cereal Chemists International (AACCI) Official Method 32-07.01 [22] and AOAC 991.43 [21]. The proximate composition of flour was provided by Shawnee Milling Co. (Shawnee, OK, USA). The particle size of CCPP flour was analyzed with AACCI method 66-20.01 [22] to obtain particles lower than 150 µm. Flour blends were thoroughly mixed, and their rheological behavior determined by AACCI Approved Method 54-21.02 [22] to obtain water absorption at 500 Brabender Units (BU) of consistency, development time, stability time, and mix tolerance index during mixing using a Farinograph-E (C.W. Brabender Instruments, South Hackensack, NJ, USA). High values of development and stability times are key indicators of dough strength.

#### Caffeine Concentration in CCPP

Caffeine was analyzed by high-performance liquid chromatography (HPLC) Shimadzu (Model LC-20AD/T LPGE Kit Shimadzu Co., Kyoto, Japan) using the method of the International Organization for Standardization (ISO) 20481 [23], and an aqueous extraction of 1 ± 0.001 g of CCPP in 250 mL of water. The mixture was heated in a water bath for 20 min at 90 °C and filtered through 0.20 µm nylon membrane (SUNSri, Wilmington, NC, USA). The mobile phase was 24% methanol and 76% water (*v*/*v*) at a flow rate of 1.0 mL/min, and the injection volume was 10 µL, using a RP-18 (Reverse Phase) column with isocratic elution and the absorbance recorded at 272 nm with an UV detector. The caffeine content was calculated with anhydrous caffeine (C_8_H_10_O_2_N_4_) as external standard (Fermont Chemical Company, Monterrey, Nuevo Leon, Mexico).

### 2.3. Sample Preparation for Rheology Test

#### 2.3.1. Gluten Samples Preparation

According to AACCI Approved Method 38-12.02 [22], the extraction of wet gluten was carried out with a Glutomatic System (Perten Instruments AB, Huddinge, Sweden) model 2200 with 20 g NaCl/L aqueous solution. Briefly, a 10-g flour sample was made into a dough; starch and other components were removed by washing the dough with the salt solution. The wet gluten obtained was at its intrinsic water-binding capacity.

#### 2.3.2. Dough Sample Preparation

The dough was prepared at constant water absorption using the value of flour without CCPP obtained in the farinograph. Samples were prepared in a modified Mixograph (National Manufacturing, Lincoln, NE, USA) equipped with a 10 g bowl and mixed for 4 min.

### 2.4. Compression-Recovery, Bi-Axial Deformation

Gluten samples were shaped as previously reported [17] using a Perten Centrifuge 2015 at 2430× *g* for 1 min (Perten Instruments AB, Huddinge, Sweden). A compression-recovery based on biaxial deformation was performed using a gluten compression-relaxation (Gluten CORE) Analyzer (Perten Instruments AB, Huddinge, Sweden) with the following conditions: velocity start 20 mm/s, compression rate 4 mm/s, target force 0.5 N, target force compression 8 N, compression time 5 s, target force recovery 0.2 N, and recovery time 55 s. Gluten elastic recoverability was reported as elastic recovery in percentage.

### 2.5. Dough and Gluten Sample Loading for Creep and Recovery Test

Freshly extracted gluten and prepared dough samples with different substitution levels of CCPP were relaxed under a press of 2.5 kg top plate with a 2.5 mm gap for 40 min at room temperature as reported [13]. A 25 mm gluten and dough disc were loaded into an AR1000 rheometer (TA Instruments, New Castle, DE, USA). A parallel hatched plate (diameter = 25 mm) geometry was used with a 2.5 mm gap, and the temperature was kept constant at 25 °C. The gluten and dough discs were retrimmed, and the edges covered with mineral oil to prevent moisture loss during the test. The constant stress of 100 Pa was applied for 100 s in the creep phase, and the strain was recorded in the recovery phase (without stress) during 100 s to measure the deformation response of gluten and dough samples (within the linear viscoelastic region).

### 2.6. Modeling of Viscoelastic Properties of Bread Dough and Gluten

An analog mechanical model composed of the concepts of springs (Hookean bodies) and dashpots (fluid bodies) was used to study the viscoelastic behavior of bread dough and gluten. The Kelvin–Voigt generalized model of six elements was used to describe experimental data:

(1) $$\gamma \left(t\right)=\frac{\sigma_{0}}{G_{0}}+\frac{\sigma_{0}}{G_{1}}\left(1-e^{\frac{-t}{\lambda_{1}}}\right)+\frac{\sigma_{0}}{G_{2}}\left(1-e^{\frac{-t}{\lambda_{2}}}\right)+\frac{\sigma_{0}}{\eta_{0}}t$$

where *γ*(*t*) is the strain as a function of time, *σ*_0_ is the applied stress, *G*_0_ is the elastic modulus for the Hookean body response: *G*_1_ and *G*_2_ are elastic moduli of the first and second retarded elastic response (first and second Kelvin–Voigt elements, respectively). *η*_0_ is the steady state viscosity [13]. *λ*_1_ = *η*_1_/*G*_1_ and *λ*_2_ = *η*_2_/*G*_2_ are the first and second retardation time required to achieve 63.21% of deformation in the expected creep of the specific Kelvin–Voigt element. The *η*_1_ and *η*_2_ are the viscous response of the first and second retarded elastic deformation, respectively, of the Kelvin–Voigt element’s dashpot. In terms of shear compliance:

(2) $$J\left(t\right)=\frac{\gamma \left(t\right)}{\sigma_{0}}=J_{0}+J_{1}\left(1-e^{\frac{-t}{\lambda_{1}}}\right)+J_{2}\left(1-e^{\frac{-t}{\lambda_{2}}}\right)+\frac{t}{\eta_{0}}$$

where *J*(*t*) is the compliance (1/Pa), *J*_0_ is the compliance at zero time, *J*_1_ and *J*_2_ are the compliances in the first and second retarded elastic response, respectively. After removing the shear stress, the recovery curve was analyzed with a similar equation for recovery compliance *Jr*(*t*); as there is no viscous flow in the recovery phase, the equation only has five elements [13].

(3) $$Jr\left(t\right)=\frac{\gamma \left(t\right)}{\sigma_{0}}=Jr_{0}+Jr_{1}\left(1-e^{\frac{-t}{\lambda r_{1}}}\right)+J_{r2}\left(1-e^{\frac{-t}{\lambda r_{2}}}\right)$$

where *Jr*(*t*) is the compliance (1/Pa) at the recovery phase, *Jr*_0_ is the compliance at zero time at the recovery phase, *Jr*_1_ and *Jr*_2_ are the compliance in the first and second retarded elastic response at the recovery phase and *λr*_1_, *λr*_2_ are the first and second retarded elastic response at the recovery phase.

### 2.7. Bread Baking Quality

AACC Approved Method 10-10.03 [22] for optimized bread-baking test for evaluating wheat flour quality was carried out with 1-pound loaves. Loaf height, weight, and volume were measured 1 h after baking. Volume was measured by rapeseed displacement following Approved Method 10-05.01 [22]. Bread crumb firmness was measured on days 1, 4, and 7 of storage at room temperature (25 °C) according to the American Institute of Baking (now AIB International) Standard Procedure for White Pan Bread [24] using 25% of height compression in two bread slices of 12.5 mm thickness. A 25 mm round probe and 10 g force of compression were used.

### 2.8. Statistical Analysis

Creep recovery curves were fitted with Origin Pro 9.1 software (OriginLab, Northampton, MA, USA) using nonlinear regression analyses. Analysis of variance, multiple means comparison (*p* < 0.05, Tukey), and Pearson’s correlations were performed with JMP Pro 14.0.0 (SAS Institute Inc., Cary, NC, USA).

## 3. Results and Discussion

### 3.1. Wheat Flour and CCPP Analysis

**Figure A1:**
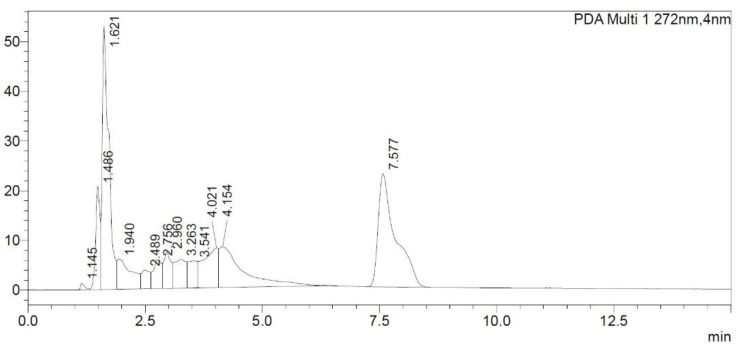
Chromatogram for caffeine, caffeine peak at 7.58 min.

Table 1 contains the proximate and caffeine analyses of CCPP and wheat flour. CCPP total dietary fiber was 44% (10% soluble fiber and 34% insoluble fiber) and caffeine 0.04% (Appendix A Figure A1, shows an example of a chromatogram for caffeine content analysis). The CCPP substitutions yielded flour blends with a significant increase in dietary fiber (by 22, 44, and 89%) and ash (by 3.6, 7.1, and 14.3%) for 1.25, 2.5, and 5% levels, respectively, compared to wheat flour control.

### 3.2. Water Absorption and Mixing Properties

Table 2 shows farinograph results of doughs with different levels of substitution of CCPP. The percentage of water absorption (WA) for dough formation increased by 1.7% at the last level of CCPP substitution (5%) compared to the control. Changes in the water absorption are due to the increase in fiber content; the flour with 5% of CCPP had a 4.5% increase in total dietary fiber. These results agree with other reports with similar trends when wheat bran [25] and commercial insoluble tomato fiber [26] were added to wheat flour. Anil [6] suggested that this was explained by hydroxyl groups in the structure of fiber-forming more interaction with water via hydrogen bonds by adding hazelnut fiber in bakery products. The development and stability times are values that indicate the dough’s strength; high values suggest strong doughs. In contrast to Anil’s results [6], the addition of CCPP significantly decreased development and stability times (Tukey, *p* < 0.05) by 30 and 48%, respectively. Our results agree with Boita and collaborators [25], who reported a significant decrease in development time and stability, and increased mixing tolerance index with wheat bran. This may be explained in part by fiber hindering the formation of disulfide bonds between wheat proteins resulting in reduced molecular size [27], weakening their cohesion, and decreasing gluten development. The highest Mixing Tolerance Index (in BU) is on samples with the higher concentration of added CCPP (5%). High values of Mixing Tolerance Index are related to weak doughs [28].

### 3.3. Compression-Recovery

**Figure 1:**
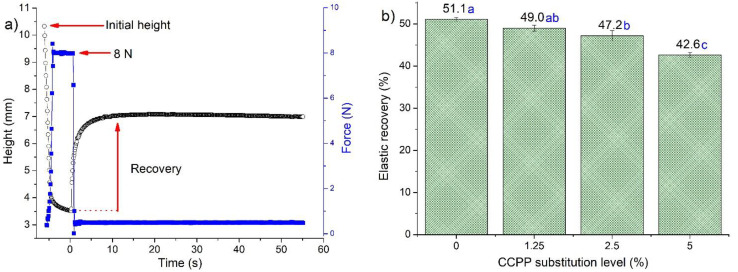
The elastic recovery of gluten from dough containing coffee cherry pulp powder (CCPP). (**a**) Typical compression-recovery curve. (**b**) Means (columns with standard error bar) with the same letter are not significantly different (*p* < 0.05, Tukey).

Figure 1 shows results from compression-recovery test for the extracted gluten of doughs with different CCPP substitution levels. The elastic recovery, calculated as the ratio of the total height recovered to the compressed height [29], decreased significantly (*p* < 0.05) by 4, 8, and 17% (1.25, 2.5, and 5% of CCPP, wet basis, respectively). Changes of greater magnitude in gluten elastic recovery compared to the mixing farinograph parameters suggest a larger effect of fiber in gluten than dough. Appendix B Table A1 shows the Pearson’s correlation coefficients, with their respective *p*-values, between the rheological and bread quality parameters. Elastic recovery was positively correlated (*r* = 0.91, *p* < 0.0001) with bread volume.

### 3.4. Creep-Recovery Test

Table 3 shows the estimated regressed parameters of the generalized Kelvin–Voigt model for the creep and recovery tests of dough and gluten with different substitution levels of CCPP. The fitting of experimental creep data by the Kelvin–Voigt model has an average *R*^2^ of 0.96. The pure elastic component (*G*_0_) in the creep phase is 1.7 and 2.8 times higher for wet gluten and dough, respectively, when 5% of CCPP was added to flour, compared with the controls. Similar trends were found by Mironaesa and collaborators [15] using grape peel fiber of different sizes on wheat dough rheology, in which the compliance associated with the pure elastic element decreased. Another study evaluated the effect of incorporating tomato seed flour in dough samples, finding that the pure elastic element’s compliance decreased with the increased addition of tomato seed flour [15]. These observations can be explained in part by the reduction in disulfide bonds and molecular size of high molecular size aggregates, as reported with the addition of fiber to gluten and gluten–starch systems [27]. On the other hand, Hernandez-Estrada and collaborators [13] reported that non-gluten components mainly affect the purely elastic component, which provides firmness to the matrix.

**Figure 2:**
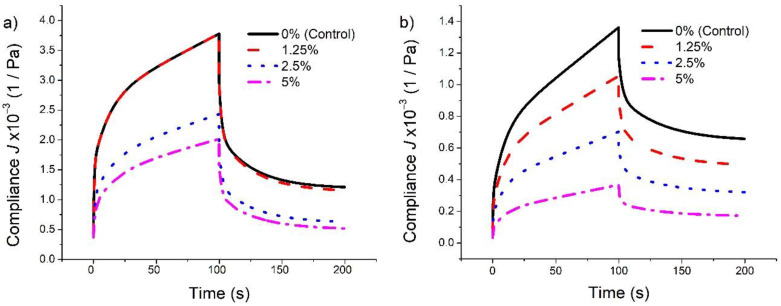
Creep-recovery curves for extracted gluten (**a**) and dough (**b**) with different levels of coffee cherry pulp powder (CCPP) substitution plotted from averages of estimated regressed parameters of Kelvin-Voigt model. 0, 1.25, 2.5, and 5% of CCPP wet basis.

*G*_1_ and *G*_2_ values increased 1.8 and 1.6 times, respectively, for gluten. A bigger increase was observed for *G*_1_ and *G*_2_ values for dough, which increased 3.6 and 3.8 fold, respectively, at 5% CCPP (wet basis) substitution compared to the control. The steady state viscosity increased 1.7 times when the CCPP substitution reached 5%. A higher value on elastic moduli indicates that more stress is needed to deform the sample, and, therefore, samples with higher substitution levels are stiffer than control. This can be seen graphically in Figure 2, where the curves were built with the Kelvin–Voigt model’s average values. Samples with higher substitution levels have lower compliance and higher resistance to deformation than other levels. It was observed in both gluten and dough. The maximum strain in dough decreased 73% with 5% of CCPP compared to the control, in contrast to 41% decrease in gluten. Maximum deformation was lower in gluten compared to that of dough, and this could be attributed in part to a possible portion of the fiber that was solubilized and removed during the isolation of gluten, as suggested by other researchers [30]. Interestingly, the curves for gluten control and 1.25% CCPP overlapped while this was not observed in dough (Figure 2). The CCPP fiber might have more interaction with the carbohydrate fraction than the protein fraction, and the maximum strain can be evidence of that, as well as 1.25% has not reached the limit of measurable differences in gluten deformation.

When the average elastic moduli (*G*_0_, *G*_1_, and *G*_2_) were compared, the dough presented a value two times higher than gluten since starch provides stiffness to the dough, as indicated in other reports [13]. The changes in dough deformation were inversely related to the CCPP levels used. As the level of CCPP increased, the deformation decreased as observed in an increase in elastic moduli, suggesting stiffer dough. Similar trends were reported by other authors [16]. Comparing the recovery magnitude: gluten recovered 1.4 times more than dough since gluten proteins provide the elasticity (recoverability capacity) and starch the dough’s stiffness. CCPP levels did not affect the recovery of gluten in the creep recovery test. Besides, the CCPP level in dough only affected its recovery at the 2.5 level compared to the control. The lack of a trend on recovery by fiber addition has been reported [15,16].

Besides significant differences in maximum strain and their respective reduction with CCPP fiber (Table 3), gluten and dough preserved their remarkable spring back to return to their original shape in the creep and recovery test conditions. In contrast, when analyzed with a large deformation performed with a compression recovery test using 8 N, the gluten recovery showed a significant decreasing trend. It suggests that a large deformation is more selective within this study and the levels of CCPP (Figure 1). The stiffness increase could be related to the water retention properties of the CCPP fiber, which possibly decreases the free water in gluten and dough, decreasing particle movements in the structure.

### 3.5. Bread Volume and Crumb Firmness

**Figure 3:**
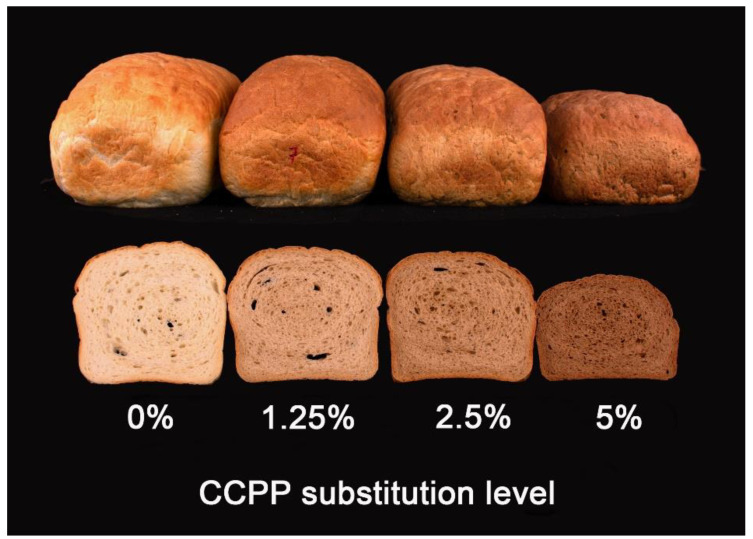
Bread loaves with different levels of coffee cherry pulp powder (CCPP) substitution.

Table 4 shows the breadmaking parameters obtained with the AACCI standard method 10-10.03 [22]. As the substitution levels of CCPP increased (1.25, 2.5, and 5% of CCPP wet basis), the bread loaf volume decreased (8.7, 21, and 36%, respectively). Compliance values of creep-recovery test were positively correlated (*r* = 0.90, *p* < 0.0001), with bread volume, in contrast to the elastic moduli (*G*_0_, *G*_1_, *G*_2_), and the steady state viscosity (*η*_0_) in the dough (*r* = −0.95) and gluten (*r* = −0.93), which were negatively correlated (*p* < 0.001) with bread volume (Appendix B). A significant decrease in bread volume was observed even at the lowest substitution level (1.25% CCPP). A low concentration of CCPP also affected the dough’s rheological properties, such as weakening of the gluten network and increasing the system stiffness. That implies less expansion of dough with CCPP substitution treatments compared to the control. Wheat flour substitution by CCPP has a similar effect on bread volume as that reported for whole grain flour and the incorporation of dietary fiber from different sources. Dietary fiber has been attributed to dilute the protein network and weaken the gluten matrix formation [5,6,25,26,31]. A qualitative darkening of bread crust and crumb increased as the CCPP substitution level increased (Figure 3). Dark bread color is commonly reported with the addition of fiber [6]. This is also common in most specialty bread loaves whose characteristics are expected to differ from most white flour bread loaves.

**Figure 4:**
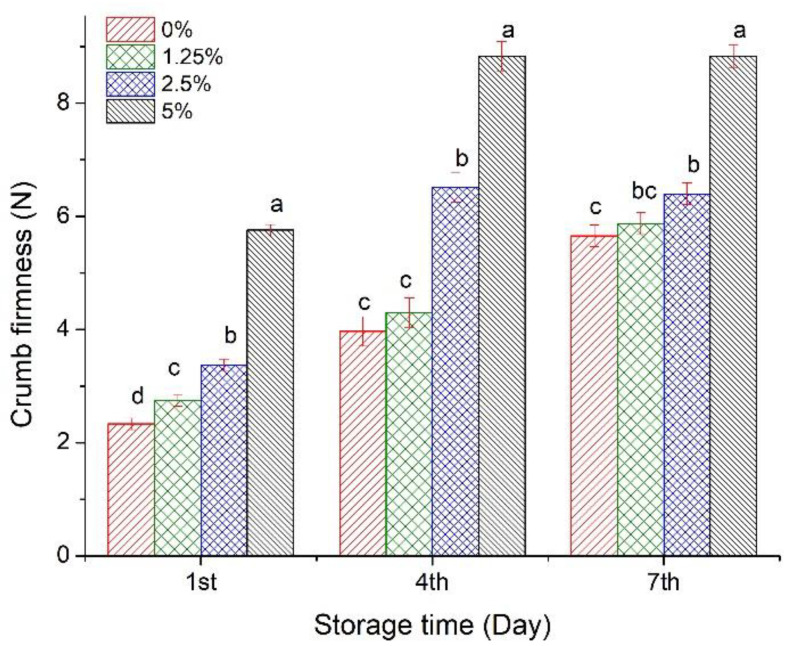
Bread crumb firmness as a function of coffee cherry pulp powder substitution level grouped by storage time. Means (columns with standard error bar) with the same letter and within the same storage day group are not significantly different (*p* < 0.05, Tukey).

Figure 4 shows the crumb firmness results according to the AACCI standard procedure 74-09.01 at the 1st, 4th, and 7th day of storage. Firmness increased significantly 59% at the maximum substitution level on the 1st day of storage. These results agree with a 54% increase in crumb firmness with cane sugar bagasse fiber was substituted at 5% in wheat flour [32]. The firmness increased 55 and 36% on the 4th and 7th day of storage, respectively. The results show an adverse effect of CCPP on crumb firmness and bread volume due to high fiber content. Sakhare and Prabhasankar [33] reported that fenugreek fiber addition decreased volume and significantly increased crumb firmness. Our results agree with trends on bread crumb firmness increase by bran reincorporation [25]. Some authors suggest that fiber interferes with the starch–gluten matrix resulting in a weakened dough to capture fermentation gas bubbles, decreasing bread volume, and increasing crumb firmness [20,25,33].

## 4. Conclusions

The partial substitution of wheat flour by CCPP affects the rheology of the dough and gluten. The water absorption in the farinograph increased by 1.7% in the 5% CCPP substitution level. The elastic recovery of gluten, compression recovery test, decreased when the CCPP levels increased and it was positively correlated (*r* = 0.91, *p* < 0.0001) with bread volume. Similarly, there was an increase in the elastic moduli and the steady state viscosity in both gluten and dough due to the increased fiber addition. In this case, both were negatively correlated (Appendix B) with bread volume (*r* = −0.93, *p* < 0.0001). The firmness of the crumb also increased when CCPP increased. This first approach has been performed without dough or bread additives (emulsifiers, enzymes, etc.), so more research should be done using dough and bread improvers to take advantage of the coffee pulp in the baking process. Consumer acceptance evaluation is suggested using the maximum level, in the case of this study was 5%, but higher levels might be achieved using dough and bread improving agents and masking flavors for the earthy after taste at higher levels of substitution. At 5% CCPP substitution, dietary fiber increased by 89% compared to the control. Our results demonstrate that the mechanical model used can quantitatively represent dough and gluten’s viscoelastic properties with CCPP and their correlation to bread volume.

## Figures and Tables

**Table 1:** Coffee cherry pulp powder (CCPP) and wheat flour proximate and caffeine analyses *.

Compounds	CCPP (g/100 g)	Wheat Flour (g/100 g)
Moisture	6.1 ± 0.0	12.6
Protein	9.7 ± 0.1	11.7
Dietary fiber	44.5 ± 0.6	2.37
Ash	4.2 ± 0.1	1.09
Lipids	1.5 ± 0.0	<1.0
Caffeine ***	0.04 ± 0.0	n.a.
Total carbohydrates **	78.7	73.5

* Means of two independent analysis. n.a. = not analyzed. ** Total carbohydrates were calculated by difference. *** Appendix A Figure A1.

**Table 2:** Mixing properties and water absorption of flour blends *.

CCPP Substitution Level (% Wet Basis)	Water Absorption (%)	Development Time (min)	Stability Time (min)	Mixing Tolerance Index (BU)	Time to Break Down (min)
0 (Control)	62.1 ± 0.1 a	11.0 ± 1.7 a	16.3 ± 1.7 a	19.7 ± 3.2 a	18.9 ± 1.7 a
1.25	62.2 ± 0.1 a	9.4 ± 0.5 ab	9.8 ± 0.8 b	39.7 ± 4.0 b	12.5 ± 0.8 b
2.5	62.6 ± 0.1 b	8.4 ± 0.1 ab	8.7 ± 0.2 b	53.0 ± 2.6 c	11.1 ± 0.4 bc
5	63.8 ± 0.1 c	7.7 ± 0.3 b	8.2 ± 0.1 b	63.3 ± 5.7 c	9.8 ± 0.3 c

* Mean values (*n* = 3 ± standard deviation) within a column followed by the same letter are not significantly different (*p* < 0.05, Tukey).

**Table 3:** Estimated regressed parameters of the Kelvin–Voigt model for extracted gluten and dough with coffee cherry pulp powder (CCPP) substitution *.

Substitution Level	Gluten				Dough			
	0%	1.25%	2.5%	5%	0%	1.25%	2.5%	5%
**Parameters**	**Creep Phase (Gluten)**				**Creep Phase (Dough)**			
*G*_0_ (Pa) × 10^3^	1.5 ± 0.1 c	1.5 ± 0.1 c	2.2 ± 0.1 b	2.6 ± 0.2 a	10.8 ± 0.4 c	13.1 ± 1.3 c	17.8 ± 0.7 b	30.6 ± 2.1 a
*G*_1_ (Pa) × 10^3^	1.2 ± 0.1 c	1.2 ± 0.1 c	1.7 ± 0.1 b	2.1 ± 0.2 a	4.8 ± 0.3 d	6.3 ± 1.0 c	8.9 ± 0.6 b	17.3 ± 0.9 a
*G*_2_ (Pa) × 10^3^	1.1 ± 0.1 c	1.0 ± 0.0 c	1.5 ± 0.1 b	1.7 ± 0.1 a	7.1 ± 0.5 c	9.2 ± 1.9 c	13.5 ± 0.9 b	26.6 ± 2.3 a
*η*_0_ (Pa) × 10^5^	1 ± 0.1 a	1 ± 0.1 a	1.4 ± 0.1 b	1.7 ± 0.1 c	1.5 ± 0.1 a	2.1 ± 0.3 a	3.2 ± 0.1 b	6.1 ± 0.5 c
Max strain (%)	34.2 ± 2.3 a	34.3 ± 0.8 a	24.0 ± 1.3 b	20.1 ± 1.7 c	13.4 ± 0.8 a	10.4 ± 1.8 b	6.9 ± 0.4 c	3.6 ± 0.3 d
	**Recovery Phase (Gluten)**				**Recovery Phase (Dough)**			
*Gr*_0_ (Pa) × 10^3^	1.3 ± 0.1 c	1.3 ± 0.1 c	1.8 ± 0.1 b	2.2 ± 0.2 a	5.5 ± 0.1 c	6.9 ± 0.8 c	9.5 ± 0.6 b	17.5 ± 1.3 a
*Gr*_1_ (Pa) × 10^3^	1.1 ± 0.1 c	1.1 ± 0.1 c	1.6 ± 0.1 b	1.9 ± 0.2 a	4.0 ± 0.1 c	5.2 ± 0.8 c	7.9 ± 0.6 b	16.6 ± 1.1 a
*Gr*_2_ (Pa) × 10^3^	1.1 ± 0.1 c	1.1 ± 0.0 c	1.6 ± 0.1 b	1.8 ± 0.2 a	3.5 ± 0.2 c	4.5 ± 0.7 c	6.4 ± 0.3 b	12.1 ± 0.8 a
Final strain (%)	8.4 ± 0.6 a	8.0 ± 0.7 a	5.9 ± 0.7 b	5.0 ± 0.5 b	6.3 ± 0.5 a	4.8 ± 1 b	3.0 ± 0.1 c	1.6 ± 0.2 d
Recovery (%)	75.4 ± 2.3 a	76.7 ± 2.3 a	75.6 ± 2.1 a	74.9 ± 2.1 a	53 ± 1.4 b	54 ± 1.9 ab	55 ± 0.6 a	54 ± 1.2 ab

* Mean values (*n* = 5 ± standard deviation) in the same row and same material (gluten or dough) followed by different letter are significantly different (*p* < 0.05, Tukey). Creep test at 100 Pa of shear stress for 100 s; *G*_0_ = instantaneous shear modulus; *G*_1_, *G*_2_ = retarded shear moduli; *η*_0_ = steady state viscosity; *Gr*_0_, *Gr*_1_, *Gr*_2_ stands for instantaneous and retarded shear moduli respectively in the recovery phase. 0, 1.25, 2.5, 5% (wet basis) of CCPP substitution levels.

**Table 4:** Bread performance parameters *.

CCPP Substitution Level (% Wet Basis)	Height (mm)	Weight (g)	Volume (cm^3^)	Specific Volume (cm^3^/g)
0 (Control)	127 ± 5 a	736 ± 4 a	3056 ± 108 a	4.14 ± 0.1 a
1.25	118 ± 3 a	739 ± 3 a	2804 ± 87 b	3.79 ± 0.1 b
2.5	106 ± 5 b	735 ± 12 a	2408 ± 98 c	3.28 ± 0.1 c
5	91 ± 2 c	737 ± 1 a	2007 ± 45 d	2.72 ± 0.1 d

* Mean values (*n* = 2 ± standard deviation) within a column followed by different letter are significantly different (*p* < 0.05, Tukey).

## Abbreviations

The following abbreviations are used in this manuscript:

CCP	Coffee cherry pulp
CCPP	Coffee cherry pulp powder
CONACyT	National Council for Science and Technology
HPLC	High Performance Liquid Chromatography
WA	Water absorption
MTI	Mixing Tolerance Index
ER	Elastic recovery
USDA	United States Department of Agriculture

## Appendix B. Pearson’s Correlation

**Table A1:** Pearson’s correlation coefficients (*r*) between traditional quality indicators and elastic recovery with creep parameters of dough and gluten *^x^*.

Gluten												
Parameters	*J*_0_	*G*_0_	*J*_1_	*G*_1_	*λ*_1_	*J*_2_	*G*_2_	*λ*_2_	*η*_0_	*η*_1_	*η*_2_	ER
Development time	0.64 *	−0.69 *	0.66 *	−0.69 *	ns	0.73 **	−0.72 **	ns	−0.73 **	−0.66 *	−0.70 *	0.76 **
Stability time	0.64 *	−0.63 *	0.65 *	−0.63 *	ns	0.71 *	−0.66 *	ns	−0.66 *	−0.60 *	−0.64 *	0.72 **
Firmness (Day 1)	−0.86 **	0.90 ***	−0.85 **	0.89 ***	ns	−0.85 **	0.87 **	0.65*	0.87 **	0.89 ***	0.89 ***	−0.89 **
Mixing tolerance	−0.89 ***	0.88 **	−0.90 ***	0.87 **	ns	−0.90 ***	0.87 **	0.62*	0.85 **	0.87 **	0.88 **	−0.87 **
Weight	ns	ns	ns	ns	ns	ns	ns	ns	ns	ns	ns	ns
Volume	0.94 ***	−0.94 ***	0.94 ***	−0.93 ***	−0.57 ^+^	0.92 ***	−0.92 ***	−0.70*	−0.91 ***	−0.93 ***	−0.93 ***	0.91 ***
Water absorption	−0.85 **	0.90 ***	−0.85 **	0.90 ***	ns	−0.87 **	0.89 ***	0.59*	0.90 ***	0.88 **	0.90 ***	−0.91 ***
Elastic recovery	0.89 **	−0.92 ***	0.89 ***	−0.91 ***	ns	0.89 **	−0.90 ***	−0.66*	−0.89 **	−0.91 ***	−0.91 ***	1
**Dough**												
Development time	0.75 **	−0.70 *	0.73 **	−0.69 *	ns	0.70 *	−0.68 *	ns	−0.70 *	−0.69 *	−0.67 *	0.76 **
Stability time	0.78 **	−0.64 *	0.78 **	−0.63 *	0.65 *	0.76 **	−0.63 *	ns	−0.65	−0.61	−0.61	0.72 **
Firmness (Day 1)	−0.90 ***	0.99 ***	−0.87 **	0.99 ***	−0.68 *	−0.86 **	0.98 ***	ns	0.98 ***	0.97 ***	0.99 ***	−0.89 **
Mixing tolerance	−0.95 ***	0.87 **	−0.94 ***	0.86 **	−0.77 **	−0.93 ***	0.86 **	ns	0.87 **	0.85 **	0.84 **	−0.87 **
Weight	ns	ns	ns	ns	ns	ns	ns	ns	ns	ns	ns	ns
Volume	0.98 ***	−0.95 ***	0.97 ***	−0.94 ***	0.82 **	0.95 ***	−0.94 ***	ns	−0.95 ***	−0.92 ***	−0.93 ***	0.91 ***
Water absorption	−0.91 ***	0.97 ***	−0.87 **	0.97 ***	−0.72 *	−0.86 **	0.96 ***	ns	0.97 ***	0.94 ***	0.98 ***	−0.91 ***
Elastic recovery	0.89 ***	−0.91 ***	0.87 **	−0.90 ***	0.65 *	0.84 **	−0.89 **	ns	−0.90 ***	−0.88 **	−0.90 ***	1

*^x^* Creep test at 100 Pa of shear stress for 100 s. *, **, and *** are significant at *p* < 0.05, 0.01 and 0.0001, respectively; ^+^ indicates significant at *p* < 0.06; and ns = not significant. *G*_0_ = instantaneous shear modulus; *G*_1_, *G*_2_ = retarded shear moduli; *J*_0_ = instantaneous compliance; *J*_1_, *J*_2_ = retarded elastic compliance; *η*_1_, *η*_2_ = viscous response associated to retardation time *λ*_1_ and *λ*_2_, respectively; *η*_0_ = steady state viscosity; Mixing Tolerance = Mixing tolerance index, development time and stability time at 500 BU in farinograph test. ER = Elastic recovery from compression-recovery test.
